# Perforated Appendicitis With Deciduosis and Free Air Under the Diaphragm During Pregnancy

**DOI:** 10.7759/cureus.83758

**Published:** 2025-05-08

**Authors:** Masumi Kiyose, Michihisa Shiro, Mieko Inagaki, Takahiro Watanabe, Tetsuo Maeda

**Affiliations:** 1 Obstetrics and Gynecology, Chibune General Hospital, Osaka, JPN; 2 Pathology, Chibune General Hospital, Osaka, JPN; 3 Radiology, Chibune General Hospital, Osaka, JPN

**Keywords:** appendicitis, computed tomography, deciduosis, free air under the diaphragm, pregnancy

## Abstract

Appendicitis is the leading non-obstetric surgical emergency during pregnancy. Its diagnosis is complicated by pregnancy-related anatomical changes and poses significant risks for maternal and fetal mortality and morbidity. This report describes a rare case of perforated appendicitis with deciduosis and free air under the diaphragm during pregnancy. A 28-year-old woman presented with severe right lower and middle abdominal pain and signs of inflammation at 31 weeks of gestation. Contrast-enhanced computed tomography revealed free air under the right diaphragm with no evidence of acute perforated appendicitis or peri-appendiceal free air. Suspecting idiopathic gastrointestinal perforation, exploratory surgery was planned following an emergency cesarean section. Exploratory surgery revealed perforated appendicitis with an abscess. Histological examination revealed deciduosis of the appendicitis with a perforation. If appendicitis cannot be identified on imaging during pregnancy and free air is present under the diaphragm, it is necessary to consider the possibility of perforated appendicitis with deciduosis, in addition to idiopathic intestinal perforation.

## Introduction

Appendicitis is the most common cause of non-obstetric surgery during pregnancy [1]. The incidence of appendicitis during pregnancy has been reported to be 0.1%-0.2% [1]. The preoperative diagnosis of appendicitis is more challenging in pregnant women than in non-pregnant women due to anatomical changes [2]. Furthermore, appendicitis is associated with a high risk of maternal and fetal mortality and morbidity due to intra-abdominal inflammation [3].

Gastrointestinal perforations present with severe abdominal pain and signs of peritonitis, most commonly indicated by intra-abdominal free air, and can be diagnosed using computed tomography (CT). However, the incidence of perforated appendicitis presenting with intra-abdominal free air detected on CT is relatively low, with one study reporting a rate of 8% [4]. In cases of acute perforated appendicitis, free air is usually detected on CT scans in the peri-appendiceal area [5]. Cases of acute perforated appendicitis with free air under the diaphragm are rare and, to our knowledge, have not previously been reported in pregnant women.

Deciduosis is defined as the presence of ectopic decidual tissue outside the uterus [6]. It is most commonly observed in the ovaries [7] and cervix [8]. Appendiceal deciduosis typically occurs during pregnancy [9]. Unlike other organs, appendiceal deciduosis may cause symptoms due to progesterone exposure and uterine compression [9]. However, there have been no previously reported cases of perforated appendicitis with free air under the diaphragm caused by appendiceal deciduosis during pregnancy, and a preoperative diagnosis of appendicitis could not be made due to the limitations of imaging in the presence of the gravid uterus.

Here, we present a case of perforated appendicitis with deciduosis characterized by the unusual presence of intra-abdominal free air under the diaphragm during pregnancy.

Written informed consent was obtained from the patient for the use of their records and accompanying images.

This article was presented as a poster at the 76th Annual Meeting of the Japan Society of Obstetrics and Gynecology on April 19, 2024.

## Case presentation

A 28-year-old pregnant woman (gravida 1, para 0) with a history of surgery for endometriosis was admitted to our hospital at 31 weeks and 1 day of gestation with severe pain in her right lower and middle abdomen.

The patient was diagnosed with threatened preterm labor caused by an intrauterine infection, based on regular uterine contractions occurring every 3 min, a shortened cervical length (20 mm), and signs of inflammation. The fetus had a reassuring non-stress test (NST). The patient’s body temperature was 37.4 ℃, and blood tests revealed elevated white blood cell (WBC) count (14,300/µL) and C-reactive protein (CRP) levels (2.04 mg/L). Ampicillin and ritodrine hydrochloride were administered to manage the threatened preterm labor and suspected intrauterine infection.

**Figure 1:**
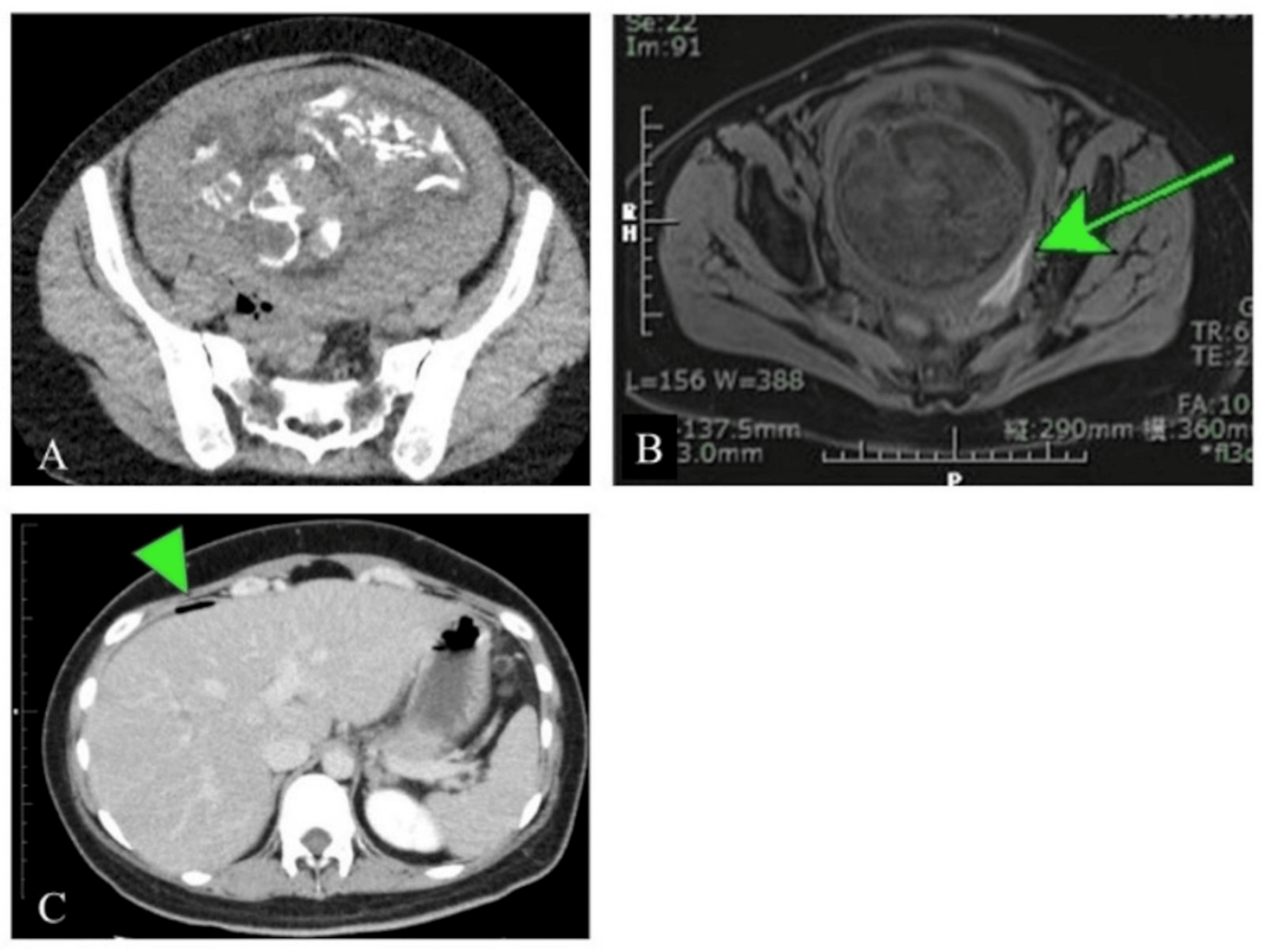
Abdominal computed tomography (CT) and magnetic resonance imaging (MRI) at 31 gestational weeks. (A) Plain CT image at 31 weeks and 1 day. The image shows no evidence of acute appendicitis. (B) T1-weighted MRI at 31 weeks and 2 days. The image reveals hemorrhagic fluid accumulation in the pouch of Douglas (arrow). (C) Contrast-enhanced CT image at 31 weeks and 2 days. The image reveals free air under the right diaphragm (triangle) with no evidence of acute appendicitis or peri-appendiceal free air.

Despite the administration of tocolytics, her abdominal pain persisted. She continued to have right lower and middle abdomen and developed rebound tenderness. Since appendicitis is the most common cause of acute abdomen during pregnancy, unenhanced abdominal computed tomography (CT) was performed. However, the initial CT scan showed no evidence of acute appendicitis (Figure 1A).

On the following day (31 weeks and 2 days), her pain progressively worsened and spread to involve the entire abdomen and back. Inflammatory marker levels further increased (WBC: 15,100/µL; CRP: 11.52 mg/L). Magnetic resonance imaging (MRI) was performed to evaluate for acute perforated appendicitis. MRI revealed pelvic peritonitis extending from the pelvic floor to both lumbar regions, as well as fluid accumulation in the pouch of Douglas, but no evidence of acute appendicitis (Figure 1B). The fluid was hemorrhagic and appeared to originate from the left posterior uterine surface.

Considering the MRI findings and the patient’s history of endometriosis, spontaneous hemoperitoneum during pregnancy was considered a possible diagnosis. A second contrast-enhanced CT scan was performed to identify the source of bleeding. This scan revealed free air under the right diaphragm, with no evidence of acute perforated appendicitis or periappendiceal free air (Figure 1C).

Suspecting idiopathic gastrointestinal perforation, exploratory surgery was planned following an emergency cesarean section. During surgical exploration, perforated appendicitis with an abscess was detected, and an appendectomy was performed. The　appendix was located in the right paracolic gutter. No evidence of perforation was observed in the small bowel or colon.

A male infant weighing 1,740 g was delivered, with Apgar scores of 4 and 7 at 1 and 5 minutes, respectively. Postoperatively, the mother received intravenous cefmetazole sodium for five days, followed by oral amoxicillin. She was discharged without complications on postoperative day 9. Apart from preterm birth, the infant's course was uneventful.

**Figure 2:**
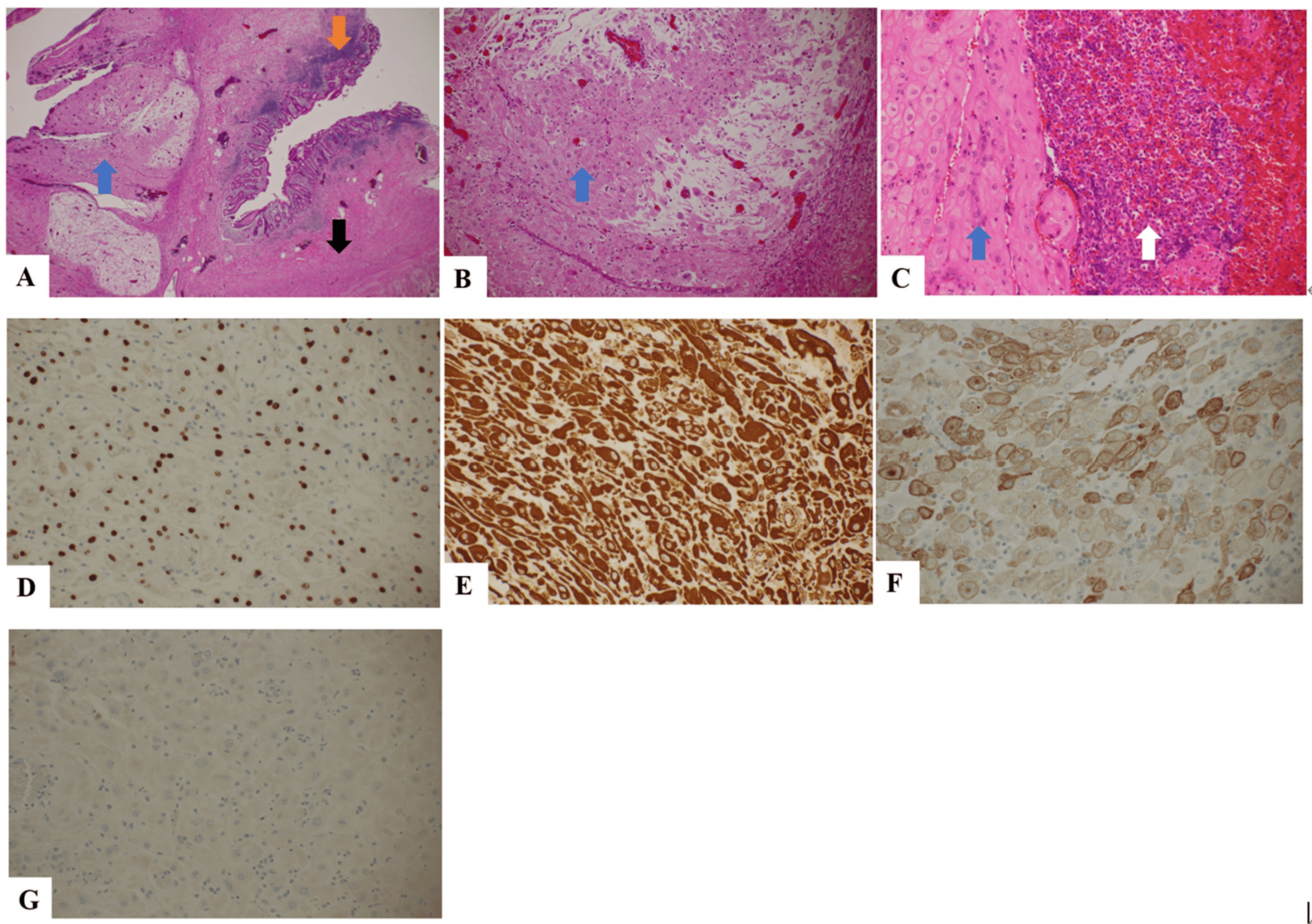
Images of histological examination of the resected appendix. (A) original magnification 20× (B) original magnification 100× (C) original magnification 200×. The smooth muscle of the appendiceal wall is completely replaced by decidual cells, with marked degeneration and loss of both the circular and longitudinal muscle layers. Severe neutrophilic infiltration is observed throughout the appendiceal wall, and an abscess involving the subserosal and serosal layers is evident. Perforation of the appendix is present, accompanied by uniform findings of suppurative peritonitis. The blue arrow indicates decidual cells, the orange arrow indicates the mucosa, the black arrow indicates the smooth muscle layer, and the white arrow indicates the abscess. (D-G) Images from immunohistochemical analysis: (D) positive for progesterone receptor, (E) positive for vimentin, (F) positive for CD10, and (G) negative for AE1/AE3.

Histological examination of the resected appendix revealed that the smooth muscle of the appendiceal wall was completely replaced by decidual cells (Figure 2). Severe deciduosis caused structural degeneration at the base of the appendix, which likely resulted in perforation. An abscess involving the subserosa and serosa of the appendix was evident, and acute inflammation was confirmed. Immunohistochemical analysis showed that the decidual cells were positive for progesterone receptor, vimentin, and CD10, and negative for estrogen receptors and AE1/AE3. No endometrial glands were detected in the appendix. Appendiceal deciduosis was diagnosed pathologically.

## Discussion

In the present case, the diagnosis of appendicitis was difficult due to the gravid uterus. While the presence of free air may suggest mediastinal emphysema, rupture of an intra-abdominal abscess caused by gas-forming organisms, traumatic perforation, iatrogenic injury, or idiopathic gastrointestinal perforation; however, idiopathic gastrointestinal perforation, the most common cause, was initially considered the most likely diagnosis. Appendicitis involves luminal obstruction at the base of the appendix, leading to ischemia of the appendiceal wall and subsequent bacterial translocation across the compromised mucosa, resulting in transmural inflammation [10]. The most common etiology of appendicitis is stercolith obstruction. The amount of extraluminal air in this condition is generally small or absent, usually no more than 1-2 mL, and free pneumoperitoneum is rare in patients with perforated appendicitis [11].

In the present case, since the abdominal CT image showed free air under the diaphragm, and CT and MRI did not identify appendicitis, we initially suspected gastrointestinal perforation of the small intestine or colon. The inability to identify appendicitis due to an enlarged gestational uterus and the assumption that free air under the diaphragm is not seen in appendicitis led to the denial of appendicitis and suspicion of other gastrointestinal perforations.

Perforated appendicitis with deciduosis is reportedly caused by obstruction of the appendix due to the proliferation of ectopic decidual tissue [12]. Other causes include high concentrations of prostaglandins, which act as potent muscle stimulants in deciduosis, contracting the appendicular muscle wall and causing appendicitis [12,13].

Typical obstructive appendicitis caused by stercoliths makes it unlikely for intestinal gas from the ileum to flow due to the obstruction of the appendix lumen by stercoliths. In contrast, in perforated appendicitis due to deciduosis, no stercoliths are present, and intestinal gas may escape through the hole after perforation.

Even if the gas leaks out through the perforated appendicular foramen, it is difficult for it to spread to the surrounding area because of the large gestational uterus. In this case, the initial abdominal CT scan did not reveal any free air under the diaphragm; it was first identified on the second CT scan that was conducted the following day. The reason for the delay in the appearance of free air under the diaphragm was that the gas that leaked into the abdominal cavity spread slowly because of the enlarged gestational uterus.

Appendicitis is more difficult to diagnose on imaging in pregnant women than in non-pregnant women. Basaran and Basaran [14] reported pooled sensitivity and specificity estimates for CT in pregnant patients with prior normal or inconclusive ultrasonography findings: sensitivity was 85.7% (95% CI: 63.7%-97%) and specificity was 97.4% (95% CI: 86.2%-99.9%), with positive and negative likelihood ratios of 10.1 (95% CI: 3.4-30.1) and 0.21 (95% CI: 0.05-0.88), respectively. For MRI in similar patients, the study [14] also reported pooled sensitivity and specificity of 80% (95% CI: 44%-98%) and 99% (95% CI: 94%-100%), with positive and negative likelihood ratios of 22.7 (95% CI: 6.0-87.5) and 0.29 (95% CI: 0.13-0.68), respectively.

In this case, perforated appendicitis with deciduosis could not be diagnosed despite the use of both CT and MRI. This suggests that imaging might have some limitations in diagnosing appendicitis with deciduosis because the cause of appendicitis is not obstructed by stercoliths. If clinical examination findings strongly suggest perforated appendicitis despite negative CT and MRI results, emergency exploratory laparotomy should be considered as a diagnostic and therapeutic option, considering the possibility of appendicitis with deciduosis.

## Conclusions

This case underscores the diagnostic challenges posed by appendicitis with deciduosis during pregnancy, particularly in the presence of atypical imaging findings such as free air under the diaphragm. The rare nature of appendiceal deciduosis, combined with pregnancy-related physiological changes, can obscure diagnosis, as observed in this patient.

If appendicitis cannot be identified on imaging during pregnancy and free air is present under the diaphragm, it is necessary to consider the possibility of perforated appendicitis with deciduosis, in addition to idiopathic intestinal perforation.
